# Mitochondrial Signaling: Forwards, Backwards, and In Between

**DOI:** 10.1155/2013/351613

**Published:** 2013-05-29

**Authors:** Sean P. Whelan, Brian S. Zuckerbraun

**Affiliations:** Department of General Surgery, University of Pittsburgh and VA Pittsburgh Healthcare System, F1200 PUH, 200 Lothrop Street, Pittsburgh, PA 15206, USA

## Abstract

Mitochondria are semiautonomous organelles that are a defining characteristic of almost all eukaryotic cells. They are vital for energy production, but increasing evidence shows that they play important roles in a wide range of cellular signaling and homeostasis. Our understanding of nuclear control of mitochondrial function has expanded over the past half century with the discovery of multiple transcription factors and cofactors governing mitochondrial biogenesis. More recently, nuclear changes in response to mitochondrial messaging have led to characterization of retrograde mitochondrial signaling, in which mitochondria have the ability to alter nuclear gene expression. Mitochondria are also integral to other components of stress response or quality control including ROS signaling, unfolded protein response, mitochondrial autophagy, and biogenesis. These avenues of mitochondrial signaling are discussed in this review.

## 1. Introduction

Since the first observations of mitochondria in the mid to late 1800s, our understanding of their structure and function has evolved significantly. The first half of the 20th century saw the characterization of the mitochondrion as the major source of energy leading to its epithet, “the powerhouse of the cell.” This paved the way for localization of the respiratory chain and TCA cycle components, as well as the confirmation of the oxidative phosphorylation hypothesis in the following years. Mitochondria were found to have DNA, RNA, and protein synthesis capabilities, and seminal investigations into mitochondrial function in yeast led to an improved understanding of mammalian mitochondrial biogenesis [1].

Nuclear factors governing mitochondrial biogenesis and function have been extensively studied over the past several decades leading to the discovery of an array of nuclear respiratory factors, hormone receptors, and important transcription factor coactivators that collectively influence mitochondrial biogenesis, oxidative phosphorylation, fatty acid *β*-oxidation, and reactive oxygen species production among a myriad of other effects [2].

In 1987, Parikh et al. investigated changes in nuclear gene expression in response to mutations in mitochondrial DNA (mtDNA) in yeast [3]. This and subsequent studies utilizing genome wide transcriptomic analyses identified target genes likely involved in a signal transduction pathway from mitochondria to the nucleus termed the retrograde pathway, which includes the retrograde response genes: RTG1, RTG2, and RTG3. Though this retrograde signaling pathway is centered on glutamate homeostasis, it has since been implicated in a number of other processes such as mitochondrial DNA maintenance, autophagy, and cellular longevity. 

Concurrently, a progressive appreciation of mitochondrial function (and dysfunction) in metazoans has implicated the mitochondrion in pivotal roles in bioenergetic homeostasis, metabolic regulation, innate immunity, and aging to name a few. Rho^0^ cell models (cells that are devoid of mitochondrial DNA) have shed light on the role of mtDNA and its products in cellular feedback mechanisms, and several mutations in human mtDNA have been identified that are responsible for a number of neuromuscular disorders, mostly involving defective mitochondrial t-RNA; however, mammalian homologues of the retrograde response genes are yet to be identified. As opposed to yeast, though, the heterogeneous tissues of metazoans also have a heterogeneous population of mitochondria, and energy metabolism is not uniform throughout [4, 5]. It may be, then, that a cohort of signals from mitochondria as well as a wide array of cellular responses to mitochondrial dysfunction represent a complex evolution of the collective mitochondrial retrograde signal.

This review discusses antegrade signaling from nucleus to mitochondria as well as the retrograde response in yeast. Retrograde signaling in mammalian cells along with similar stress signaling including the unfolded protein response and intermitochondrial signaling is reviewed as well. 

## 2. The Antegrade Pathway: Nuclear Contribution to Mitochondrial Biogenesis

Mitochondria are double-membraned organelles present in almost all eukaryotic cells. Endosymbiotic theory postulates that they, along with other organelles such as chloroplasts in plants, originated from free-living bacteria that were taken into cells and developed a symbiotic relationship. The evolution of this complex relationship hypothesizes that eukaryotic cells with glycolytic energy production via the nuclear genome and cytosolic machinery merged with the oxidative mitochondrion. Most of the mitochondrial genome was then transferred to the nuclear DNA. In this new complex relationship, the nuclear DNA not only encodes the genes for determining cellular and organismal structure but also the genes for glycolysis and most of the genes for oxidative metabolism [6]. The mitochondrial genome, which is maternally inherited, retains the core genes for generating, maintaining, and using the mitochondrial inner membrane potential. The epigenome then coordinates nuclear DNA gene expression based upon the environmental calories available. Therefore, the mitochondria are semiautonomous in that they depend on nuclear contribution for much of their functionality, and in fact, many of the catalytic regions of the complexes are controlled by the nucleus [7]. Furthermore, nuclear genes are responsible for the transcription factors and the transcriptional machinery required for the expression of the mitochondrial genome. Each mitochondria carries varying copy numbers—usually 2–10 copies per organelle—of their own double-stranded DNA plasmids, and due to a high mutation rate, mitochondrial DNA can vary within a single cell, and populations in organs vary based upon regional energy environments. The human mitochondrial genome consists of 37 genes: 13 for protein subunits, 22 for mitochondrial t-RNA, and two for ribosomal RNA [2].

Advances in the understanding of the molecular basis of mtDNA transcription were made with the characterization of the transcription factor, Tfam (formerly known as mtTFA) [8, 9]. It was identified as a high-mobility group (HMG) box protein involved in specific binding to enhancers upstream from bidirectional promoters in the D-loop of the mitochondrial chromosome. Tfam has also been shown to bind randomly at nonspecific sites of mtDNA prompting the suggestion that it functions to stabilize mtDNA as well. Mitochondrial polymerase has been purified in yeast and consists of a single subunit RNA polymerase (RPO41p) coupled to a specificity factor [10]. While human polymerase has not been purified, cDNA database screening has identified proteins with homology to lower eukaryotes as well as similarity to the bacteriophage polymerases T7 and T3 [11, 12]. This has been shown to act in concert with mtTFB, a protein with significant homology to prokaryotic sigma factors involved in promoter recognition. While this is also analogous to the yeast specificity factor Mtf1p, it does not confer specificity in higher eukaryotes. Both RPO41p as well as other eukaryotic polymerases initiate transcription in a nonspecific manner and require specificity factors in order to associate with the appropriate promoter region. As mentioned, Mtf1p serves this function in yeast, whereas Tfam assumes the responsibility in humans [11].

**Figure 1:**
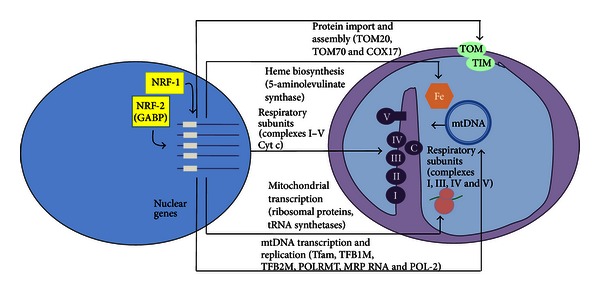
Diagrammatic summary of the nuclear control of mitochondrial functions by NRF-1 and NRF-2 (GABP). NRFs contribute both directly and indirectly to the expression of many genes required for the maintenance and function of the mitochondrial respiratory apparatus. NRFs act on genes encoding cytochrome c, the majority of nuclear subunits of respiratory complexes I–V, and the rate-limiting heme biosynthetic enzyme 5-aminolevulinate synthase. In addition, NRFs promote the expression of key components of the mitochondrial transcription and translation machinery that are necessary for the production of respiratory subunits encoded by mtDNA. These include Tfam, TFB1M, and TFB2M as well as a number of mitochondrial ribosomal proteins and tRNA synthetases. Recent findings suggest that NRFs are also involved in the expression of key components of the protein import and assembly machinery. Adapted with permission from [2].

A new set of nuclear-encoded transcription factors were discovered when Evans and Scarpulla identified recognition sites on the cytochrome c promoter with no homology to those found in yeast [13]. A transcription factor termed as nuclear respiratory factor 1 (NRF-1) was then found to have specific biding sites in the promoters of cytochrome c as well as other genes involved in the electron transport chain [14]. The scope of influence of NRF-1 along with a second nuclear respiratory factor, NRF-2, has since been expanded tremendously to include vital components of oxidative phosphorylation, the mitochondrial transcription factors Tfam and mtTFB, rate-limiting steps in heme synthesis, ion channel synthesis, and mitochondrial protein import, assembly, and shuttling [15, 16]. Other nuclear transcription factors are shown to have recognition sites in the promoters; genes encoding respiratory proteins are the estrogen-related receptor ERR*α*, which also regulates fatty acid *β*-oxidation, and the general transcription factor Sp1, which appears to be unique in its ability to both positively and negatively regulate respiratory component transcription [17]. Regulation of fatty acid *β*-oxidation represents another important layer of nuclear control. While not technically part of the respiratory apparatus, this pathway is used by mitochondria to generate acetyl CoA as a carbon source for the TCA cycle and ultimately provide substrate for the electron transport chain. In addition to regulation by ERR*α*, peroxisome proliferator-activated receptors (PPAR*α* and PPAR*δ*) perform this function, though in contrast to ERR*α*, they do not seem to influence transcription of respiratory proteins [2] (Figure 1).

Finally, a family of coactivators has been shown to interact broadly with these distinct nuclear respiratory transcription factors, potentially providing a means of coordination or fine-tuning. The most prominent of these is the peroxisome proliferator-activated receptor gamma, coactivator 1-alpha (PGC1*α*) [18]. First recognized for its interaction with PPAR*γ* in adipocyte differentiation, PGC1*α* responds to a complex set of physiologic signals to activate NRF1, NRF2, Tfam, mtTFB, ERR*α*, PPAR*α*, and all the attendant sequelae culminating in mitochondrial biogenesis. This coactivator, in particular, seems to represent an important link between the products of mitochondrial function or dysfunction and the subsequent alterations in nuclear gene expression [19].

## 3. The Retrograde Pathway

### 3.1. Retrograde Signaling in Yeast

 Nuclear regulation of the mitochondrial network is extensive and complex. However, the paradigm of mitochondrial signaling leading to changes in nuclear gene expression is relatively novel and is considered mitochondrial retrograde signaling. Many pathologic conditions, as well as some physiologic ones, are associated with mitochondrial dysfunction, which has become increasingly correlated with subsequent changes in nuclear gene expression. The first studies done in yeast deficient in mtDNA identified the accumulation of several RNA transcripts in the nucleus [3]. Later, transcriptomic analyses in rho^0^ cells identified a variety of nuclear-encoded transcripts that were increased [20, 21].

Liao et al. recognized that the CIT2 gene, encoding the peroxisomal citrate synthase (CIT2), was consistently and dramatically upregulated in rho^0^ cells and became regarded as the prototypical target of the retrograde pathway in yeast [22]. CIT2 plays an important role in citrate synthesis as part of the glyoxylate pathway in peroxisomes as opposed to the mitochondrial citrate synthase enzyme, Cit1. Glutamate is the only nitrogen source for biogenesis in yeast and is derived primarily from the *α*-ketoglutarate generated in the TCA cycle. The TCA cycle is disrupted in respiratory-deficient cells, and peroxisomal anaplerotic contributions become critical to maintenance of an adequate pool of *α*-ketoglutarate. Identification of regulators of the CIT2 gene led to discovery of several retrograde response (RTG) genes. Four positive and at least four negative regulators of CIT2 have been identified [23, 24]. Subsequent microarray analyses on rho^0^ cells have suggested many areas along the TCA and glyoxylate cycles that are controlled in a similar fashion [25]. Additionally, Freije et al. used RNAi knockdown of glycolytic enzymes in *Drosophila* followed by microarray analyses to show a shift from oxidative phosphorylation to aerobic glycolysis [26]. 

Rtg2p is a cytoplasmic phosphohydrolase central to the induction of the retrograde response. Activation of Rtg2 leads to disinhibition of the downstream transcription factors, Rtg1p and Rtg3p. This is achieved by dephosphorylation of the inhibitory factor Mks1p. Partially, dephosphorylated Mks1p is also targeted for degradation by the E3 ubiquitin ligase, Grr1p. While this would seem to designate Grr1p as a positive regulator of the retrograde response, it has been suggested that its primary role involves degradation of a free pool of Mks1p making the Rtg2p-mediated regulation more efficient. Conversely, two 14-3-3 proteins, Bmh1p and Bmh2p, have been shown to interact with Mks1p preventing Grr1p-dependent degradation and, therefore, inhibiting activation of the Rtg1/3p heterodimer [27, 28]. 

**Figure 2:**
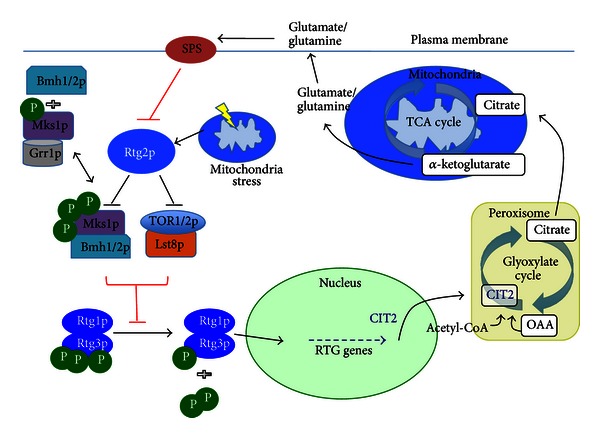
Positive and negative regulators of the retrograde pathway. The retrograde pathway is constitutively inhibited by Mks1p as well as TOR1/2p/Lst8p which hyperphosphorylates (P) the Rtg1/3p heterodimer. Bmh1/2p stabilizes the phosphorylated Mks1p contributing to its activity and preventing its degradation. Mitochondrial stress activates Rtg2p which dephosphorylates Mks1p. Mks1p then dissociates from Bmh1/2p and is degraded by Grr1p. Rtg2p also inhibits the inhibitory factor Lst8p. Additionally, Lst8p is part of the TOR1/2p complex and is also controlled by canonical regulators of TOR. The disinhibition of the Rtg1/3p heterodimer allows dephosphorylation and translocation to the nucleus where it activates the RTG genes. The prototypical RTG gene CIT2 encodes peroxisomal citrate synthase (CIT2) which converts Acetyl-CoA and oxaloacetic acid (OAA) to citrate. This contributes nitrogen to the TCA cycle in order to maintain an adequate supply of *α*-ketoglutarate. Ultimately, this leads to production of glutamate which is the ultimate source of biosynthetic reactions in yeast. The plasma membrane amino acid sensor SPS inhibits Rtg2p in a negative feedback mechanism in the presence of excess glutamate.

The TOR kinase complexes have also been shown to inhibit the retrograde response, perhaps consistent with their other functions in nutrient sensing [29]. Integral to this inhibitory effect is the Lst8p protein that is a component of the TOR1/2p complex. Glutamate has been proposed to exert a negative feedback effect on the pathway either directly or through the membrane-bound SPS amino acid-sensing complex, though absence of glutamate alone is not sufficient to inhibit retrograde target genes. An additional level of control is implicated in the observation that the Rtg2p protein contains an ATP binding domain that is required for its function. This suggests that Rtg2p may act as an ATP sensor, activating retrograde signaling in response to low ATP levels [30] (Figure 2). 

Given the role of the mitochondria in producing ATP, it is not surprising that the relative metabolic state of the cell with regard to ATP concentration would have an impact on mitochondrial activity. Amiott and Jaehning showed that each mitochondrial promoter has a unique sensitivity to mitochondrial ATP concentration and that levels of ATP have a direct relationship on the activity of mitochondrial RNA polymerase (mtRNAP) [10]. Furthermore, they suggested a role of AMP/ATP concentration in coordinating mitochondrial and nuclear gene expression via Snf1 kinase, the ortholog of mammalian AMP kinase. Overall, energy status as measured by AMP can influence retrograde signaling as well. After disruption of the electron transport chain, increases in AMP can modulate cell cycle progression [31].

### 3.2. Calcium Homeostasis and Retrograde Signaling

 Calcium signaling and homeostasis is critical to normal cell function. It is responsible for initiation of life after fertilization of the oocyte, differentiation of cells during development, intracellular and intercellular signaling, and ultimately for initiation of cell death [32]. Mitochondria have long been recognized to have important roles in calcium signaling and homeostasis and are often separated into two groups with relation to their primary function in this respect: those in excitable cells and those in nonexcitable cells [33]. Transient oscillations in calcium concentration are organized by location and amplitude and are important in transmitting intra- and intercellular signals. Mitochondrial calcium concentration is generally low due to a set point generated by the Na+/Ca++ antiporter and the Ca++ uniporter on the inner mitochondrial membrane, while most intracellular calcium is sequestered in the endoplasmic or sarcoplasmic reticulum [34]. In excitable cells, such as brain dendritic cells, cardiac myocytes, smooth muscle cells, and others, mitochondria can influence cytosolic calcium in a variety of different ways. With increasing concentrations of calcium, they can store calcium in the matrix in the form of hydroxyapatite (the main building block of bone), tricalcium phosphate, or other calcium phosphate precipitates. Therefore, mitochondria can propagate calcium-driven signals in two ways: acting as a calcium sink in order to prevent feedback inhibition or acting as a calcium reservoir releasing more calcium to the cytosol to amplify signals. Interestingly, calcium can also activate plasma membrane potassium channels to hyperpolarize a cell depressing excitability. The difference between the ultimate consequences of intracellular calcium concentration and its handling by mitochondria seems to be mostly related to the spatial relationships, length of calcium transients, and to a smaller extent to the amplitude of the oscillations. 

 A sustained elevation of intracellular calcium is associated with initiation of either necrotic cell death or the initiation of apoptotic machinery [35]. One example is glutamate excitotoxicity in neurons with overstimulation of the NMDA receptor followed by prolonged cytosolic calcium elevation. Mitochondria undergo the membrane permeability transition with swelling of the matrix and rupture of the outer membrane. If all mitochondria are affected, cell necrosis ensues; however, when enough mitochondria are functional to sustain ATP production after caspase activation and cytochrome c release, apoptotic cell death follows. 

 Given the importance of calcium in cell processes and the function of the mitochondrion in calcium signaling, it is not surprising that calcium would play a key role in mitochondrial retrograde signaling (also called mitochondrial stress signaling). This can be inferred by the well-described membrane permeability transition in response to mitochondrial stress associated with ΔΨm depolarization, the appearance of the permeability transition pore, and calcium efflux. It has also been seen in experiments analogous to those done in yeast in which nuclear transcriptional analysis of mammalian rho^0^ cells is investigated [20].

### 3.3. Retrograde Signaling in Mammalian Cells

Though mammalian orthologues of the Rtg proteins have not been identified, some target genes of mammalian mitochondrial retrograde signaling have been described [36]. Alteration of mtDNA in several cell lines generally resulted in mitochondrial membrane depolarization and increased cytosolic calcium leading to increased transcription of genes regulating calcium homeostasis. Amuthan et al. cultured human pulmonary adenocarcinoma A549 cells in the presence of ethidium bromide to selectively and partially inhibit mtDNA replication in a similar fashion to their prior work in C2C12 rhabdomyoblasts [37]. They were able to show mtDNA depletion results in 2-3-fold increase in steady state cytosolic calcium. Calcineurin, ERK1, and ERK2 were increased resulting in nuclear translocation of transcription factors such as NFATc- and JNK-activated ATF2. Nuclear targets involved in calcium transport and storage were also induced including the ryanodine receptors (RyR1/RyR2), calreticulin, and calsequestrin. Additionally, antiapoptotic proteins Bcl2 and Bcl-X_L_ were elevated, and proapoptotic proteins Bid and Bax were decreased. Though a number of nuclear-encoded mitochondrial proteins containing CRE sequences have been found to be elevated in mitochondrial dysfunction, Arnould et al. identified CaMKIV-induced CREB phosphorylation as a new component of the retrograde pathway with Vankoningsloo et al. later adding C/EBP homologous protein (CHOP) as well [38, 39]. These results were reversed with restoration of mtDNA and were inhibited in these and other models when calcium was removed from the system. Biswas et al. provided an important link between mitochondrial stress and NF*κ*B activation in a manner distinct from canonical regulation by TNF*α*, IKK*α*/*β*, and I*κ*B*α* [40]. They showed that genetic (mtDNA depletion) or metabolic (CCCP addition) mitochondrial stress results in calcineurin-dependent inactivation of I*κ*B*β*, allowing NF*κ*B/Rel translocation to the nucleus. It has been suggested that as an organism increases in complexity, NF*κ*B takes over more of the responsibilities of stress signaling. Though NF*κ*B has no close homology to the Rtg proteins, Srinivasan et al. showed strong homologies in pathways common to both [41].

In addition to increasing cytosolic calcium concentration, mitochondrial stress has been shown to produce excess reactive oxygen species (ROS). When this stimulus overwhelms the resident antioxidant defense consisting of the superoxide dismutases, catalases, and glutathione peroxidases among others, it can result in lipid peroxidation, activation of the permeability transition pore, and apoptosis [42]. In contrast to the catastrophic consequences of overwhelming oxidant stress, though, ROS have been shown to be important second messengers in physiologic and pathologic conditions. Mitochondria can communicate among themselves via ROS second messengers as described below, and ROS can be part of an important retrograde signal by stimulating the antioxidant response element (ARE) of cytoprotective genes. One notable example is nuclear factor- (erythroid-derived 2-) related factor 2 (Nrf2). Nrf2 resides in the cytoplasm and is constitutively degraded by Keap1. In the presence of ROS, Keap1 undergoes a conformational change releasing Nrf2 that is then translocated to the nucleus [43]. There, it binds the ARE of genes involved in the antioxidant response like heme oxygenase and inducers of mitochondrial biogenesis such as NRF-1. Formentini et al. recently showed that overexpression of ATPase inhibitory factor 1 (IF1) in a colon cancer cell line was associated with mitochondrial-induced ROS-mediated retrograde signaling [44]. The elaboration of ROS was required for activation of the canonical NF*κ*B pathway and resulted in cell proliferation- and Bcl-X_L_-mediated resistance to drug-induced cell death. 

 Much of the work dedicated to deciphering the mammalian retrograde response has utilized cancer cell lines reflecting the fact that the mitochondrial defects have been associated with many types of cancers since the initial description of “aerobic glycolysis” in cancer cells by Warburg. A number of mitochondrial and nuclear DNA defects affecting genes involved in mitochondrial metabolism are associated with prosurvival or invasive properties [45, 46]. Correia et al. showed that infiltrating astrocytomas had a marked decrease in mtDNA copy number that was associated with increased levels of mitochondrial polymerase catalytic subunit and the mitochondrial transcription factors Tfam and mtTFB1/2 [47]. Wallace provides an excellent review [48], outlining multiple examples of mitochondrial genetic and metabolic defects leading to altered nuclear gene expression and tumorigenic progression.

## 4. Additional Mechanisms of Mitochondrial Retrograde/Stress Signaling

### 4.1. Mitochondrial Unfolded Protein Response (mtUPR)

**Figure 3:**
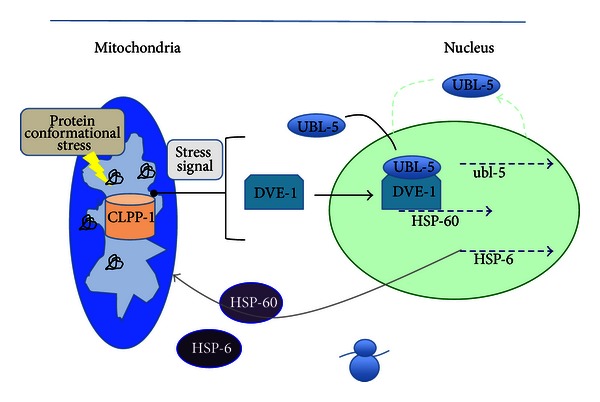
Hypothetical model of the *C. elegans *UPR^mt^ pathway. Protein conformational stress in the mitochondrial matrix triggers CLPP-1 proteolysis of an unknown substrate, producing a stress signal (blue line). The stress signal is conveyed to the cytoplasm and induces nuclear translocation and complex formation of UBL-5 and DVE-1, as well as binding of DVE-1 to the promoter of the chaperone target gene, HSP-60. This stress-signaling pathway results in the induction of mitochondrial chaperone genes, HSP-60 and HSP-6. ubl-5 expression is also upregulated, which in turn amplifies the UPR^mt^ signal (green-dotted line). HSP-60 and HSP-6 are imported into the mitochondria, where they help to restore protein homeostasis by refolding rogue proteins. Adapted with permission from [51].

The complex compartmentalization of mitochondrial networks and different sources of protein synthesis require coordination of protein import/export, folding, and proper integration. Prior identification of the roles of the cytosolic heat shock response as well as the endoplasmic reticulum unfolded protein response (erUPR) in intracellular protein homeostasis led to the characterization of the mitochondrial unfolded protein response in a similar function. To that end, several chaperone proteins were identified to play an important role in mitochondrial protein homeostasis [49]. These included the HSP-60 and HSP-70 family proteins, which in *C. elegans* are represented by hSP-60 and hSP-6, respectively. By using *C. elegans* reporter constructs in which green fluorescent protein expression was coupled to the promoter elements of hSP-60 and hSP-6, Haynes et al. identified nuclear genes important to the mtUPR. CLPP-1 is a protein homologous to the *E. coli* protease ClPP [50]. It localizes to the mitochondria and is important for initiation of the UPR (Figure 3). Additionally, the transcription factor DVE-1 was seen to interact with the promoters of the chaperone genes as well as with the ubiquitin-like protein UBL-5, which could potentially act as an amplification signal similar to the amplifying signals seen in the erUPR. While CLPP-1 is necessary for DVE-1 localization to the nucleus, the messengers between the two compartments have yet to be elucidated [51].

Although mammalian homologues of these components have not yet been identified, mammalian mtUPR target genes have been identified and include chaperonin 60, chaperonin 10, mtDNAJ, and ClPP. Additionally, these targets are upregulated in absence of induction of stress proteins involved in canonical erUPR or the cytosolic heat shock response implying some specificity to the pathway. One target common to the UPRs is the transcription factor C/EBP homologous protein (CHOP) [52]. While in the erUPR, one result of CHOP is the induction of apoptosis; its function in mtUPR is not yet clear, though there is evidence to suggest that it is involved in adaptive, prosurvival pathways [53]. For example, mtUPR in mammalian tumor cells has been associated with protective roles promoting cancer cell survival. Siegelin et al. showed that mtUPR in murine glioblastoma cells was dependent on HSP-90 and associated with tumor cell survival and adaptation [54]. Inhibition of the mtUPR by antagonizing HSP-90 resulted in apoptosis and prevention of tumor growth. In breast cancer cells, the mtUPR is activated in response to protein accumulation in the intermembrane space, and ROS production activates estrogen receptor alpha (ER*α*) [55]. This results in activation of NRF1 and other cytoprotective responses to overcome the mitochondrial stress. Additionally, disruptions in unfolded protein responses in both ER and mitochondria have been implicated in the development of neurodegenerative diseases such as Parkinson's and Alzheimer's diseases among others [56, 57].

### 4.2. Intermitochondrial Signaling

 If the mitochondrion has been shown to be a dynamic organelle, the mitochondrial network is anything but static. Spatial and temporal organization of mitochondria has been shown to be varied and complex. Most studies involving mitochondrial network dynamics have been done in cardiac myocytes, which typically have a tightly packed, lattice-like arrangement. Mitochondria are the primary source of intracellular ROS, and progressive oxidative stress leads may potentially lead to depolarization of the mitochondrial membrane potential (ΔΨm). The mitochondrial permeability transition (MPT), mediated by the permeability transition pore (PTP), is a central event in bioenergetic failure and mitochondria-initiated apoptosis and is regulated by the redox state of the mitochondrion among several other factors including calcium flux as mentioned above [58]. Perturbations in the physiologic oscillations ΔΨm can lead to progressive mitochondrial dysfunction. Zorov et al. described a method of intermitochondrial communication during oxidative stress termed ROS-induced ROS release (RIRR) in which local oxidative stress leads to release of the superoxide radical (O_2_^−^) [59]. Superoxide acts as a messenger between mitochondria leading to a wave of membrane depolarization and further ROS release. Neighboring chains of mitochondria appeared to cooperate in reversible waves of depolarization. Zhou et al. used live cardiac myocytes coupled with a mathematical model of RIRR to show that O_2_^−^ is the specific mediator of the wave of depolarization and that a reversible change in ΔΨm spread progressively in a spatiotemporal diffusion until a critical threshold was reached leading to global depolarization [60]. Park and Choi further suggested that differing spatial relationships between mitochondria in differing tissues potentially lead to alterations in primary messenger (O_2_^−^ versus H_2_O_2_) of RIRR as well as the effectiveness of different antioxidants on propagation [61].

Multiple different mechanisms of RIRR have since been described ranging from direct mitochondrial-generated ROS to complex ROS generation secondary to antioxidant inhibition or ROS-induced injury [62]. As a consequence, intermitochondrial ROS signaling is a system by which mitochondrial network dynamics can be coordinated in response to a complex system involving the myriad stimuli leading to oxidative stress and the antioxidant response system.

### 4.3. Mitochondrial Autophagy, Mitoptosis, and Biogenesis

As a corollary to retrograde and intermitochondrial signaling, autophagy is a form of quality control through interorganellar signaling. Autophagy is an evolutionarily conserved process of removing or recycling damaged organelles by engulfing them in a double-membraned autophagosome that is then taken to a lysosome for degradation [63]. Cells can maintain quality control of organellar function through a baseline level of autophagic activity [64]. In times of stress, however, the autophagic machinery can be upregulated in order to maintain cellular function by preventing the accumulation of nonfunctioning, potentially toxic organelles. Mitophagy, or macroautophagy that specifically involves mitochondria, is an important component of this process since nonfunctioning mitochondria can be particularly toxic through their generation and release of ROS and reactive nitrogen species (RNS) [65]. Unmitigated oxidative stress can lead to cell death through necrosis or apoptosis. Mitophagy is an adaptive process that is initiated through complex and incompletely understood signaling in order to prevent persistent cell damage and ultimate cell death [63, 66]. 

Another mechanism by which cells can eliminate damaged mitochondria is mitoptosis [67]. During cellular energy crises in which mitochondrial stress leads to impaired oxygen utilization, ROS production increases resulting in fragmentation of the mitochondrial network, clustering of damaged mitochondria in the perinuclear region, incorporation into a single-membraned mitoptotic body, and finally extrusion of the mitoptotic body via exocytosis or blebbing [68]. It is not clear whether this mechanism acts independently or in concert with autophagy. Lyamzaev et al. found that mature mitoptotic bodies were not associated with autophagosome, and suggested that in the setting of whole-cell energy catastrophe, mitoptosis may be a faster mechanism of mitochondrial clearing than mitophagy [68].

Once damaged organs have been cleared, a new population of mitochondria will need to be generated. The physical basis for this is, in part, the raw materials harvested through the autophagic recycling of damaged organelles. The physiological basis for the new population is mitochondrial biogenesis. Biogenesis may be initiated at the same time as autophagy or secondary to subsequent autophagic signaling. A number of signals such as ROS, calcium, energy status, and others influence the activation of the aforementioned nuclear coactivator PGC1*α* and the associated nuclear respiratory factors [69–71]. In this way, mitochondrial homeostasis is restored, and the cell is able to avoid bioenergetic failure and death.

### 4.4. Mitochondria and the Innate Immune System

The innate immune response relies heavily on ROS production in phagocytes by NADPH oxidase for bactericidal capability [72]. Recently, mitochondrial-generated ROS have been shown to contribute to macrophage bactericidal activity in response to activation of cell surface Toll-like receptors (TLR1, TLR2, and TLR4) [73]. These TLRs activate the signaling adaptor and tumor necrosis factor-associated factor 6 (TRAF6) that translocates to the mitochondria. It then ubiquitinates the protein evolutionarily conserved signaling intermediate in Toll pathways (ECSIT) that is localized to the mitochondria and is involved in complex I assembly. This leads to interruption of the respiratory chain, migration of mitochondria to the phagosomes, and increased production of mitochondrial ROS. 

### 4.5. Mitochondria and Longevity

Reactive oxygen species have long been implicated in aging, senescence, and cancer, and since mitochondria are a main source of ROS, they are often regarded as prime targets for modulation of aging [74, 75]. Use of traditional antioxidants to this end has been largely unsuccessful, perhaps because they are not reaching the appropriate compartment. More recently, however, mitochondrial-targeted antioxidants have shown some promises in this regard [76, 77]. Murphy et al. developed a system of targeting antioxidants such as ubiquinone to lipophilic cations that would preferentially migrate to the relatively negatively charged mitochondrial matrix [78–80]. Additionally, this is a rechargeable antioxidant in that it can regenerate a reduced form by accepting electrons from the respiratory chain. 

Skulachev et al. have expanded on this idea by creating SkQ-type antioxidants [81]. One drawback of these constructs is a tendency to act as prooxidants at higher concentrations. SkQ antioxidants are composed of a penetrating ion (“Skulachev ion”-Sk) and a plastoquinone (Q), which is used in place of ubiquinone, and have shown higher efficiency and lower prooxidant activity than previous compounds. In an expansive project, Skulachev et al. were able to show beneficial effects of SkQ antioxidants in multiple areas associated with tumorigenesis and aging [77, 81]. SkQ reduced cancer development in p53-deficient mice; it prevented age-related changes of retinopathy and cataracts in multiple mammalian models, and it increased life span in a number of models including the fungus *Podospora*, the crustacean *Ceriodaphnia*, *Drosophila*, and mice.

The retrograde response has also been implicated in aging and longevity [82]. Senescent mitochondria develop progressive genetic instability. In yeast, this is manifested by accumulation of extrachromosomal ribosomal DNA circles (ERC) [83]. Though activation of the retrograde response has been shown to induce ERC formation in some cases, continued activity of the retrograde response appears to contribute to longevity and prevent further genomic instability. Additionally, yeast replicative life span is dependent on a concept of age asymmetry in which mitochondrial dysfunction is not inherited in the daughter cells during division [84]. Damaged mitochondria are segregated in the mother cell with the daughter cells receiving a complement of normal mitochondria thereby conferring the capacity for a normal life span [85].

## 5. Conclusion

 Mitochondria require nuclear input in addition to their own genetic information and are aptly considered semiautonomous structures. One could also argue the reverse. Increasingly, mitochondria are seen to control nuclear gene expression as well as function and even fate of the cell. Improved understanding of mitochondrial signaling and metabolism provides significant potential to impact future of diagnosis and therapy in a wide array of physiology and pathophysiology.
